# The effects of exercise on antenatal depression: a systematic review and meta-analysis

**DOI:** 10.3389/fpsyt.2024.1290418

**Published:** 2024-09-23

**Authors:** Zheng Zhang, Yun Jia Liu, Lin Sun, Xiao-Dong Zhao

**Affiliations:** ^1^ College of Sports Science, Kyonggi University, Suwon, Republic of Korea; ^2^ Wuhan Institute of Martial Arts, Wuhan Sports University, Wuhan, China; ^3^ College of Business Administration, Kookmin University, Seoul, Republic of Korea

**Keywords:** depression, antenatal, exercise, systematic review, meta-analysis

## Abstract

**Objective:**

The aim of this study was to conduct a systematic evaluation of exercise interventions’ effectiveness on antenatal depressive symptoms in pregnant women and to investigate the impact of different intervention factors on the relationship between exercise and antenatal depressive symptoms.

**Methods:**

We conducted comprehensive searches in several databases, including PubMed, Embase, Web of Science, Cochrane Library, and others. The search period covered from database inception to May 2023. After thorough screening, a total of 7 papers with 524 subjects were included in the analysis.

**Results:**

The meta-analysis revealed that the overall effect size of exercise intervention on antenatal depressive symptoms in pregnant women was SMD = -0.41, with a 95% confidence interval of [-0.78, -0.05], and P = 0.03, indicating a significant improvement in depressive symptoms due to exercise intervention during the antenatal period. However, some degree of heterogeneity was observed among the studies, with I² = 74%, P = 0.0007.

**Conclusion:**

The results indicate that exercise interventions significantly contribute to the improvement of antenatal depressive symptoms in pregnant women, as inferred from the combined findings of the studies. Notably, static exercise intervention showed better results than dynamic exercise intervention. Moreover, interventions conducted before 20 weeks’ gestation had superior outcomes compared to those conducted after 20 weeks’ gestation, and interventions lasting longer than the trimester duration exhibited more favorable effects than shorter interventions. However, to validate these findings and optimise exercise intervention protocols for better antenatal maternal depressive symptom management, larger sample sizes and more comprehensive studies are required, given the observed heterogeneity and potential limitations in the present study.

**Systematic review registration:**

https://www.crd.york.ac.uk/prospero/ PROSPERO, identifier (CRD42023422315).

## 1 Background

The antenatal period marks a pivotal phase in a woman’s life, profoundly influencing her physical and mental well-being, and consequently impacting the trajectory of her child’s life. Nevertheless, approximately 20 per cent of women globally experience depression during this critical period of pregnancy (WHO 2017). The symptoms of antenatal depression share similarities with general depression, but it is distinct from postpartum depression. Antenatal depression exerts detrimental effects on both the maternal health and the developing foetus, heightening the risks of preterm birth and low birth weight. Symptoms of antenatal depression encompass a spectrum that includes mood swings, anxiety, sleep disturbances, and cognitive impairments, alongside negative perceptions of pregnancy. Notably, individuals may also suffer from persistent fatigue and bodily pain, coupled with diminished emotional attachment to their unborn child. These manifestations underscore the distinct clinical profile of antenatal depression, highlighting its complex interplay with both psychological and physical health parameters (1). Additionally, it may exert enduring impacts on the child’s behavioral and cognitive development (2, 3).

Numerous investigations have explored various antenatal interventions for depressed pregnant women, including antidepressants, psychotherapy, complementary and alternative medicine (CAM), and physical activity (4, 5). Antidepressant medications, encompassing selective serotonin reuptake inhibitors (SSRIs), tricyclic antidepressants (TCA), and serotonin-norepinephrine reuptake inhibitors (SNRIs), have demonstrated their efficacy in alleviating depressive symptoms and ameliorating the psychological well-being of antenatal depression patients. However, concerns have arisen regarding the potential risks to foetal development and birth defects associated with regular antidepressant use during pregnancy, prompting some women to discontinue treatment (6). Furthermore, individual differences may result in adverse effects such as dizziness, nausea, and insomnia in certain pregnant women taking antidepressants (7).

Psychotherapy, acknowledged for providing emotional support to individuals with antenatal depression, fostering feelings of empathy and care, has emerged as a viable option. Psychotherapy represents a pivotal treatment modality for antenatal depression, serving to bolster emotional support while fostering empathy and nurturing care. Various psychotherapeutic approaches, including cognitive behavioural therapy (CBT), interpersonal therapy (IPT), and psychodynamic therapy, have been rigorously evaluated and applied in this context. CBT targets maladaptive cognitive processes and behaviours, aiming to enhance mood regulation and resilience (8). IPT, on the other hand, concentrates on enhancing interpersonal relations and bolstering social support networks to address and ameliorate relational conflicts (9). Psychodynamic therapy delves into the deeper realms of the unconscious mind and the echoes of early developmental experiences, seeking to uncover and resolve the underlying psychological roots of distress (10). Each of these therapies offers distinct mechanisms through which the complex emotional challenges of antenatal depression can be addressed, reflecting the multifaceted nature of this condition. Nonetheless, its time and cost implications can hinder access to treatment for many pregnant women (11). Additionally, studies investigating the effectiveness of elements such as probiotics, vitamins, and minerals for antenatal depression in pregnant women are currently lacking sufficient scientific evidence to substantiate their efficacy and safety (12).

Exercise is increasingly recognised as a viable non-pharmacological therapy, augmenting traditional psychotherapy and pharmacotherapy in managing prenatal depression. The simplicity and accessibility of physical activity, coupled with its broad spectrum of benefits to both physical and mental health, underscore its importance. Empirical evidence reveals that regular exercise substantially elevates beta-endorphin levels within the body. This naturally occurring peptide not only mitigates pain but also enhances positive emotions by mimicking opioid effects. The mental health benefits of exercise are mediated through several key physiological mechanisms. Notably, physical activity boosts the production of neurotrophic factors such as brain-derived neurotrophic factor (BDNF), which plays a crucial role in neuronal growth, development, and repair, thereby enhancing overall brain functionality and cognitive control. Additionally, exercise stimulates the release of pivotal neurotransmitters, including serotonin, dopamine, and norepinephrine, which are integral to mood regulation and emotional stability (13). From a psychological standpoint, engaging in physical exercise fosters an individual’s sense of control and agency, attributes reinforced through the achievement of measurable fitness goals and enhanced physical self-efficacy (14). Group exercise sessions also provide valuable social interaction, reducing feelings of isolation and strengthening interpersonal connections, which are essential for mitigating prenatal depression. Moreover, mindfulness-based physical practices such as yoga and tai chi not only involve physical exertion but also incorporate meditation and mindfulness exercises. These activities enhance present-moment awareness and emotional regulation capabilities, which are crucial for managing symptoms of antenatal depression (15). Thus, exercise emerges as a multifaceted therapy with profound implications for both preventing and alleviating prenatal depression.

In the domain of antenatal care, meticulously designed exercise interventions, customised to suit the unique characteristics and physical conditions of each expectant mother, are increasingly recognised for their potential to enhance maternal health during pregnancy. The selection of appropriate exercise modalities—such as aerobics, yoga, and Pilates—and the maintenance of moderate intensity are critical to ensuring both safety and effectiveness. Moreover, continuous engagement with healthcare providers is essential to ascertain the safety and efficacy of these exercise programs. These measures not only contribute to the alleviation of symptoms associated with antenatal depression but also hold promise for the prevention of postnatal depression, underscoring their significant role in comprehensive prenatal health strategies (16).

The reverberating benefits extend even further. The amelioration of postpartum depressive symptoms not only contributes to improved infant growth, development, and behaviour but also holds the potential for enduring positive effects (17). Consequently, by addressing mood disruptions, including anxiety, sleep disturbances, and appetite fluctuations, judicious exercise emerges as a practical, non-pharmacological avenue devoid of side effects in the prevention and treatment of maternal depression.

Numerous systematic reviews have focused on exercise interventions as a valuable means of postpartum depression recovery (18–20). However, limited attention has been given to investigating the relationship between exercise and antenatal depression. Although some studies suggest the potential efficacy of exercise in addressing depression during pregnancy, their conclusions are drawn from a restricted number of trials, which exhibit considerable heterogeneity and wide confidence intervals (21–24). Additionally, these findings are predicated on a limited pool of low- and moderate-quality trials, further contributing to the heterogeneity in sample size, experimental design, and intervention approaches (21, 23).

Current reviews predominantly center their attention on dissecting the causal disparities in the impacts of antenatal versus postnatal exercise on postpartum depression, delving into the underlying rationales found within the original literature. However, they tend to provide scant specific discourse regarding the formulation of exercise regimens. Likewise, extant meta-analyses primarily fixate on assessing the quantifiable extent of exercise interventions on maternal depression, gauging the effect size. Regrettably, these endeavours often overlook pivotal moderating variables’ determination, encompassing the nature and intensity of exercises, along with their nexus to maternal depression within the context of exercise programs (20).

Conspicuously absent in the literature is an exploration of the interplay between the constituents, intensity, and other pivotal variables underpinning exercise interventions and their nexus with maternal depression. This dearth of research has resulted in a conspicuous deficiency in practical guidance and recommendations pertinent to the construction of daily exercise intervention programs tailored for maternal depression. It is worth underscoring that the crux of enhancing the efficacy of exercise intervention for maternal depression hinges on the judicious selection of exercise type, intensity, duration, and frequency. These determinants hold sway over both the effectiveness of exercise and the safety of expectant mothers.

Regrettably, there is a paucity of international exercise guidelines tailored explicitly for maternal depression. The profound divergence in individuals’ physical capacities and health statuses underscores the paramount importance of discerning the optimal exercise modality for each individual, thereby ensuring the safe engagement of pregnant women devoid of undue physical strain.

Moreover, it is imperative to factor in the multitude of physiological and metabolic transformations that transpire within a pregnant woman’s body during gestation. These encompass shifts within the cardiovascular system, hormonal profiles, body weight, and center of gravity. Therefore, the selection of exercise modality must adroitly navigate these changes to safeguard the comfort and well-being of pregnant women.

The timing of exercise assumes critical significance in the prevention of antenatal depression (25). Ideally, commencing exercise prior to conception proves most advantageous, affording the opportunity to cultivate a robust state of physical and mental well-being. However, even in cases where exercise commences during pregnancy, it is incumbent upon expectant mothers to seek professional counsel and supervision from a healthcare provider. Additionally, vigilance must be exercised with regard to the duration and frequency of exercise, as pregnant women possess distinct characteristics warranting prudent consideration. Excessive or frequent exercise can precipitate fatigue, physical discomfort, and potentially elevate the risk of injury. Hence, exercise regimens should be characterised by a gradual, progressive approach, permitting expectant mothers to incrementally acclimatise and amplify both the duration and intensity of their exercise routines. It merits acknowledgment that different modes of physical activity exert distinct effects on the body and mind (26). Static exercises, for instance, may prove beneficial in fostering relaxation and enhancing posture, while dynamic exercises may be more instrumental in fortifying cardiorespiratory function and metabolism. By juxtaposing these two modalities, it becomes conceivable to delineate which type of exercise aligns more harmoniously with the prevention and treatment of antenatal depression, tailored to individual proclivities and requisites.

To date, a comprehensive assessment of the overall effects of exercise in alleviating antenatal depression remains lacking, as there is a scarcity of systematic reviews and meta-analyses encompassing all pertinent studies. Therefore, the need arises to synthesise existing research to comprehensively evaluate the potential effectiveness of exercise in mitigating antenatal depression. Such an analysis holds the promise of providing novel insights and methodologies to enhance maternal mental health during this critical period.

## 2 Methods

This meta-analysis was performed according to the Preferred Reporting Items for Systematic Reviews and Meta-Analysis statement (27) and the Cochrane Collaboration Handbook. The protocol was registered on PROSPERO (CRD42023422315).

### 2.1 Data sources and searches

The systematic search was conducted by two independent reviewers (ZZ and LJY) in four databases: the Cochrane Library, Embase, PubMed and Web of Science, and was designed to retrieve articles up to May 2023, with disagreements resolved by consensus and by a third reviewer (SL) in case of disagreement. Terms from the Medical Subject Headings (MeSH) and words from the text were used as follows: (“Pregnant” OR “antenatal” OR “ante-natal” OR “ante-partum” OR “prenatal” OR “pre-natal” OR “prepartum” OR “pre-partum” OR “mother” OR “maternal” OR “perinatal” OR “peri-natal” OR “peripartum” OR “peri-partum”) AND (“Depression” OR “Depressive Symptoms” OR “Depressive Symptom” OR “Symptom, Depressive” OR “Emotional Depression” OR “Depression, Emotional”) AND (“Exercise” OR “Exercises” OR “Physical Activity” OR “Activities, Physical” OR “Activity, Physical” OR “Physical Activities” OR “Exercise, Physical” OR “Exercises, Physical” OR “Physical Exercise” OR “Physical Exercises” OR “Acute Exercise” OR “Acute Exercises” OR “Exercise, Acute” OR “Exercises, Acute” OR “Exercise, Isometric” OR “Exercises, Isometric” OR “Isometric Exercises” OR “Isometric Exercise” OR “Exercise, Aerobic” OR “Aerobic Exercise” OR “Aerobic Exercises” OR “Exercises, Aerobic” OR “Exercise Training” OR “Exercise Trainings” OR “Training, Exercise” OR “Trainings, Exercise”), Specific details of the search algorithms for each database are provided in **Supplementary Material 1**.

### 2.2 Inclusion and exclusion

The study’s inclusion criteria were meticulously determined based on the PICOS principles, a rigorous evaluation framework used in Cochrane systematic appraisals. Participants (P) Healthy pregnant women with no contraindications to exercise or other major medical conditions (I) multifactorial exercise interventions with different content, intensity, duration, frequency and periodicity (28) (C), routine care or other standard antenatal activities were taken into account. The primary subject of observation (O) centred around antenatal depression, with the main indicators of depression outcomes derived from various depression rating scales. The study design (S) predominantly focused on randomised controlled trials (RCTs), which are widely recognised as the gold standard for clinical effectiveness research, ensuring a robust and reliable foundation for analysis. To maintain the study’s precision and rigor, specific literature exclusion criteria were employed. Pregnant women with depression arising from other medical conditions were excluded, as were those with alcohol and cigarette abuse. Additionally, comprehensive interventions were not considered, and studies lacking complete data or not published in English were excluded. Non-RCT studies were also not included in the analysis, further ensuring the study’s consistency and validity.

### 2.3 Assessment of risks of bias

Two reviewers (ZZ and LJY) independently conducted risk of bias assessments for the included studies, adhering to the Cochrane Collaboration guidelines. This widely accepted and comprehensive tool allowed us to evaluate the methodological quality of each eligible study in a standardised manner. The Cochrane Collaboration network provides a detailed description of risk assessment for each item, with specific criteria classified as low, high, or unclear risk.

The following key areas were evaluated to assess the risk of bias for each study: randomisation, allocation concealment, blinding of participants and researchers, incomplete outcome data, selective reporting, and other biases. Due to ethical reasons, blinding of participants can often be challenging. Each study was independently assessed by two reviewers (ZZ and LJY), and any discrepancies were resolved through discussion or consultation with a third reviewer (SL) if necessary.

The results of the risk of bias assessment, providing a transparent and comprehensive overview of the methodological quality of each included study, are summarised in **Supplementary Material 2** in a risk of bias table. When interpreting the results of the META analyses and drawing conclusions about the effectiveness of preconception exercise interventions in reducing the risk of antenatal depression, we carefully considered the outcomes of this assessment.

By adhering to the Cochrane Collaboration guidelines and employing a rigorous risk of bias assessment process, we ensured the reliability and validity of our meta-analysis. This robust evaluation of each study’s methodological quality further enhances the credibility and significance of our study’s findings and conclusions.

### 2.4 Data extraction

With a standardised form, two reviewers (ZZ and LJY) independently extracted the pertinent data from each included study, encompassing essential details such as author names, year of publication, gestational weeks, sample size of the intervention and control groups, age group characteristics of both intervention and control groups, type of intervention, intervention length, frequency, and duration, type of control group, and outcome measures.

The utilisation of a standardised form ensured consistency and accuracy in data extraction across all studies, minimising the risk of errors and enhancing the reliability of the collected information. Each reviewer diligently recorded the required data elements from the eligible studies, and any discrepancies or uncertainties were resolved through discussion or consultation with a third reviewer (SL) if necessary.

By employing this rigorous and systematic data extraction approach, we obtained comprehensive and reliable information from the included studies, forming the foundation for our comprehensive META analysis. The detailed data extracted from each study are presented in the **Supplementary Materials**, providing transparency and facilitating a thorough understanding of the primary characteristics of the studies included in our research.

### 2.5 Assessment of overall effect size

Statistical analyses were conducted using Review Manager V.5.3, and overall effect sizes were calculated based on the statistical analyses of the results from the measurement scale tests of the seven included articles. Hedge’s g standardised effect sizes were utilised for each included study to measure the intervention’s effect size, with effect sizes of 0.2, 0.4, and 0.8 indicating small, medium, and large effects, respectively. To ensure consistency and that all effect sizes were in the expected direction of the intervention, p<0.05 was considered significant.

Given the different measures of the effect of exercise on antenatal maternal depression, the standardised mean difference (SMD) was chosen as it reflects the overall intervention effect size. To synthesise the effect of physical activity on antenatal depression scores in the meta-analysis, the standardised mean difference (SMD) was calculated using the Practical Meta-Analysis Effect Size Calculator (Wilson) along with its corresponding 95% confidence intervals. A heterogeneity test was also conducted to assess the extent of differences between the included studies in describing the overall effect sizes. Heterogeneity was assessed using methods such as the Q statistic and the I2 indicator. I2 values quantitatively assessed heterogeneity, where 0% indicated no heterogeneity, ≥25% indicated low heterogeneity, ≥50% indicated moderate heterogeneity, and ≥75% indicated high heterogeneity. When I2 values indicated moderate to high heterogeneity, a random-effects model was used for data combination, and conversely, a fixed-effects model was utilised (29).

Finally, potential publication bias in the results of the meta-analysis was considered, as studies without significant results are less likely to be published than those with significant results. Due to the limited number of studies (seven) included in this meta-analysis, publication bias was manually checked using assessment criteria from the Cochrane Collaboration Guidelines (CCG) for each study. The assessment covered aspects such as randomisation, masked allocation, blinding, incomplete outcome data, selective reporting, etc. The results of the bias assessment for each study were recorded in a table or form, and two assessors (ZZ and LJY) conducted the assessment independently. In case of any inconsistencies in the assessment results, a third assessor (SL) could arbitrate through discussion, if needed, to resolve any assessment disputes.

### 2.6 Subgroup analysis of exercise intervention programmes

Subgroup analyses were performed to investigate potential sources of heterogeneity and to examine whether the effect of physical activity on antenatal depression differed between subgroups, considering different characteristics of the included studies. Specifically, we conducted subgroup analyses based on three groups: the gestational age at which the exercise intervention started, the type of physical activity (classified as static and dynamic physical activity), and the duration of the exercise intervention.

Conducting subgroup analyses of this nature empowers researchers to garner deeper insights into the multifaceted effects of exercise on antenatal depression across varying contexts. Furthermore, this approach avails the opportunity to tailor clinical recommendations to align with the distinctive needs and characteristics of diverse pregnant women. This nuanced research paradigm not only enhances comprehension of exercise interventions’ impacts on antenatal depression but also fosters the formulation of finely targeted intervention strategies.

For each subgroup, effect sizes (Hedge’s g) and their corresponding 95% confidence intervals were calculated using Review Manager V.5.3. The statistical significance of the effect sizes in each subgroup was assessed at a significance level of p<0.05. Heterogeneity tests were employed to determine whether there were significant differences between subgroups.

## 3 Results

### 3.1 Search process

**Figure 1:**
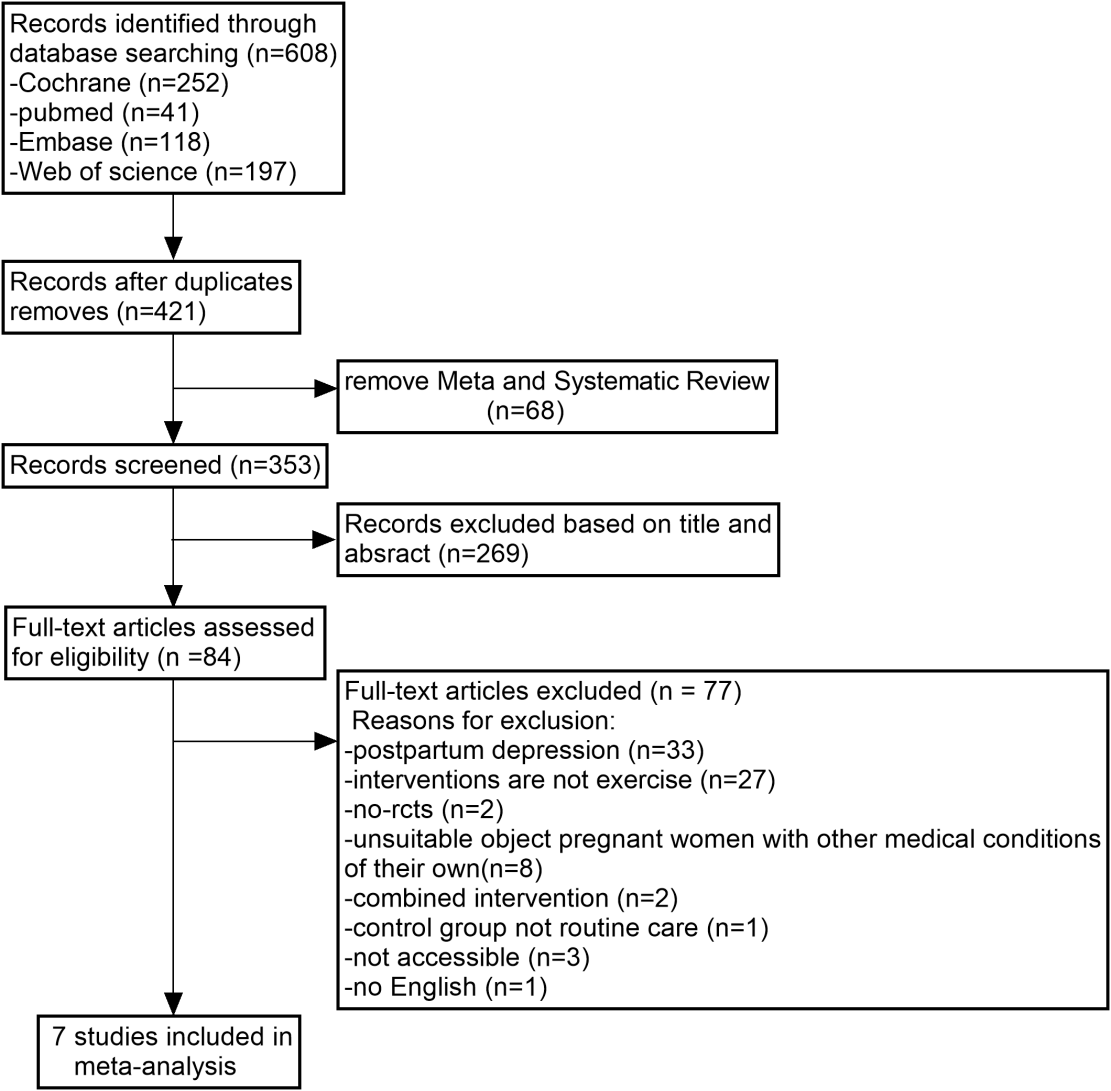
Flowchart and selection of studies.

We searched 4 databases for 608 studies. After removing duplicates, META analyses and systematic reviews were excluded from the remaining 421 studies, leaving 353 studies that were screened for titles and abstracts, resulting in 84 studies that needed to be read in full. Of these, 77 studies were excluded for reasons including researching postnatal depression, noncompliance with the intervention, and inappropriate subjects. Finally, our meta-analysis included data from 7 studies. The detailed process used to search for these is shown in **Figure 1** Flowchart and selection of studies.

### 3.2 Characteristics of the included studies and participants

The characteristics of the seven trials that were included in this META analysis are shown in **Table 1**. The publication dates of the seven studies varied from 2012 to 2022; the sample sizes of each included study varied from 19 to 167; the duration of exercise for antenatal depression in each study varied from 9 to 28 weeks’ gestation; the mean ages of the intervention and control groups are shown in **Table 1**; the types of exercise used varied from dynamic to static, the type of exercise used was a combination of aerobic exercise and a high-intensity interval training programme, the type of static exercise was yoga, and the control group was routine care and usual antenatal education. The types of exercise interventions were dynamic and static, combined aerobic exercise and high-intensity interval training for dynamic exercise, yoga for static exercise, and usual care and normal antenatal education for the control group. The duration of the exercise intervention ranged from 1 to 6 months, and the frequency of the intervention ranged from once a week to three times a week. Depression Inventory II (n=1); Centre for Epidemiological Studies Depression Scale (n=3); Hospital Anxiety Depression Scale (n=1); Profile of Mood States Questionnaire (n=1); Edinburgh Postnatal Depression Scale (n=1).

**Table 1:** Characteristics of the included studies and participants.

Studies	Sample Size(IG/CG)	Age Range(IG/CG)	Gestational weeks	IG Type	CG Type	Frequency/duration	outcome measures
Dominika Wilczyńska (2022) (30)	34/20	31 ± 4/32 ± 4	22 ± 4	HIIT	EDU group	8-week/three 60-min training sessions a week	BDI-II
Marina Vargas-Terrones (2020) (31)	36/25	32.5 ± 3.3/32.6 ± 4.7	12–16	10-min-warm-up, 25-min-aerobic, 10-min-muscle strengthening, 5-min-coordination and balance, 5-min-pelvic-floor exercises, 5–10-min-relaxation.	usual care	three sessions per week from 12–16 gestational weeks to the end of the third trimester (weeks 38–40)	CES-D
M. Satyapriya (2013) (32)	51/45	26.41 ± 3.01/ 24.96 ± 2.58	18–20	Yoga	standard antenatal exercises	2 h/day (3 days/week) for one month	HADS
M. Perales (2014) (33)	90/77	31.08 ± 3.39/ 31.66 ± 3.86	9–12	5–8-min-warm-up, 25-min- muscle strengthening, 10-min-balancing exercises, 10-min-pelvic-floor exercises, 5–8-min-relaxation.	usual care	three times per week between 9 and 12 weeks of gestation and continued Weeks 39 and 40	CES-D
Angelo Fernando Robledo-Colonia (2012) (34)	37/37	21 ± 3/ 21 ± 3	16–20	10-min-walking, 30-min-aerobic exercise, 10-min-stretching, 10-min-relaxation.	usual care	three 60-min exercise classes per week for 3 months	CES-D
Cathryn Duchette (2021) (35)	10/9	27.1 ± 2.88/30.11 ± 4.10	20.94 ± 4.69	Yoga	usual care	at least one prenatal yoga class each week for 10 weeks	POMS
Kyle Davis (2015) (36)	20/19	29.74 ± 5.40/ 30.57 ± 4.46	>28	Yoga	usual care	yoga intervention consisted of eight consecutive 75-min weekly group classes.	EPDS

IG, Intervention group; CG, Control group; HIIT , high intensity interval training program; EDU group , educational program; BDI-II, Beck depression inventory-II; CES-D, Center for Epidemiological Studies-Depression Scale; HADS, Hospital Anxiety Depression Scale; POMS, Profile of Mood States Questionnaire; EPDS, Edinburgh Postnatal Depression Scale.

### 3.3 Risks of bias

Among the seven included papers, random sequence generation was assessed as low risk in all seven studies. Five studies had a low risk of bias due to allocation concealment, while two studies had an unknown risk due to underreporting. Participant blinding was assessed as high risk in six studies and unknown risk due to underreporting in one study. Assessor blinding was rated as low risk in five studies and unknown risk in two studies. Similarly, blinding of outcome assessment was considered low risk in five studies and unknown risk due to underreporting in two studies. Regarding “incomplete outcome data,” two studies were deemed to have a low risk, while five studies were rated as unknown risk due to underreporting. Additionally, for selective reporting of study results, three studies were assessed as having low risk, and four studies were considered to have an unknown risk. Lastly, all studies were assessed as having an unclear risk of other bias due to a lack of necessary information. The comprehensive assessment of the risk of bias for each study is provided in **Supplementary Material 2**.

### 3.4 Meta-analysis result

**Figure 2:**
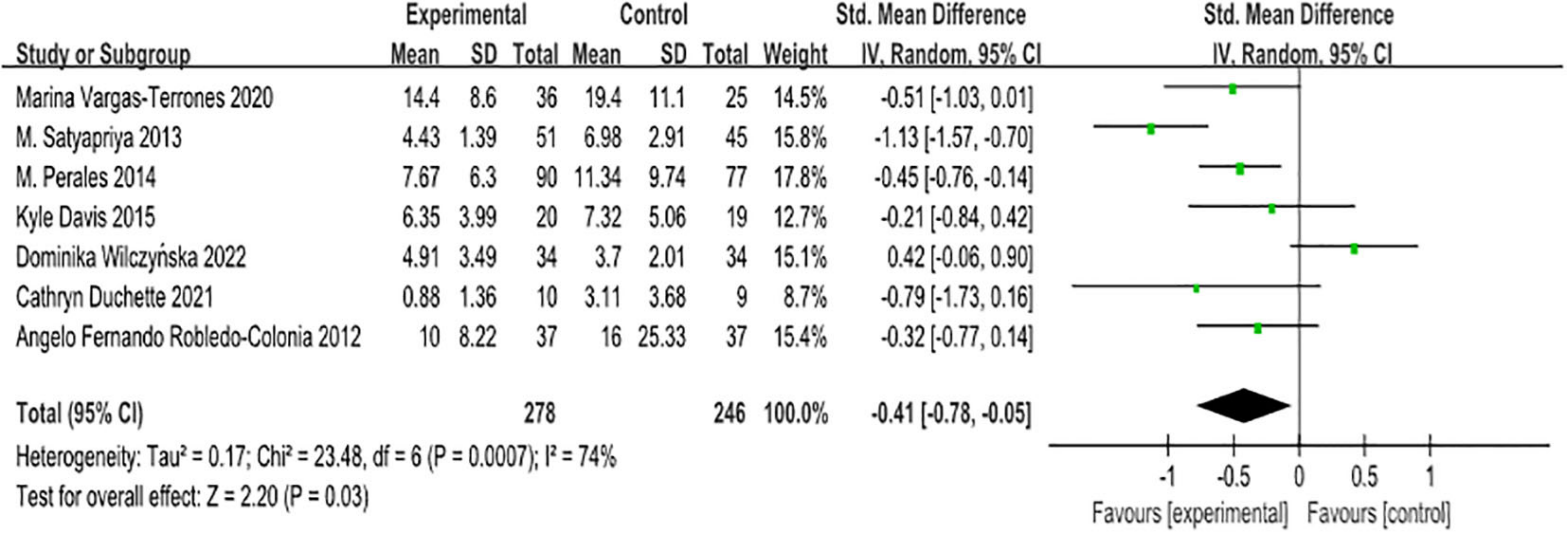
Forest plot of the effect of exercise intervention on antenatal maternal depression.

In this study, we employed various scales, namely BDI II, CES-D, HADS, POMS, and EPDS, due to their diverse measurement tools. To address this variability, the standardised mean difference (SMD) was selected for the analysis. The overall evaluation of the seven papers yielded a heterogeneity test result with df = 6 (p = 0.0007) and I² = 74%, indicating moderate heterogeneity. Consequently, we applied the random effects model to the analysis, as depicted in **Figure 2**.

The combined intervention effect size was reported as [SMD = -0.41, 95% CI -0.78 to -0.05, Z = 2.20, p = 0.03], signifying a statistically significant overall combined effect. These findings suggest that antenatal exercise can effectively alleviate depression in pregnant women. Notably, all seven papers utilised screening tools with higher scores associated with deeper depression. An effect size represented by a negative value indicates that exercise has a beneficial effect in reducing symptoms of antenatal depression in pregnant women. The SMD of -0.41 corresponds to a medium effect size, indicating that exercise can significantly improve the symptoms of antenatal depression in pregnant women.

To explore potential sources of heterogeneity in the study results, we conducted an exclusion-by-exclusion sensitivity analysis. Through this process, we systematically removed selected research literature and observed the effects on heterogeneity indicators. Interestingly, during this analysis, we observed the deletion of one study (Dominika Wilczyńska, 2022) that investigated HIIT training interventions for maternal depression. Surprisingly, the removal of this literature resulted in a significant reduction in overall heterogeneity, with a p-value of 0.08 and I² of 49%. Although the p-value did not reach the conventional significance level of 0.05, this sensitivity analysis implies that this particular literature may play an important role in contributing to the observed heterogeneity. Therefore, we have made the decision to retain this literature for further exploration and discussion, as it explores HIIT training as an intervention for maternal depression within the context of our research questions, and was published in a peer-reviewed journal (30–36).

### 3.5 Subgroup analysis of moderating variables for exercise intervention programmes

In this study, we established subgroups to examine three elements of the exercise and exercise program: the type of exercise intervention, the duration of continuous intervention, and the timing of the intervention during pregnancy (see **Table 2**). The type of exercise intervention was categorised into static exercise, represented by yoga, and dynamic exercise, represented by aerobics and HIIT. Due to the limited number of included studies and the relatively small sample size, Intervention duration is bifurcated at the threshold of three months, thereby segregating participants into two distinct groups: those with intervention durations less than or equal to three months, and those with intervention durations exceeding three months. Simultaneously, with respect to the initiation of exercise intervention, the demarcation is delineated as the commencement of pregnancy at the 20-week mark. This partitions the subjects into two categorical cohorts: those commencing intervention prior to or at 20 weeks of gestation, and those who embark on intervention after 20 weeks into pregnancy.

**Table 2:** Subgroup analysis of exercise intervention for depressive symptoms in antenatal pregnant women.

Adjustment variables	Subgroup	Heterogeneity test		Sample size	SMD [95%CI]
		P	I^2^(%)		
Type of Exercise Intervention	Static exercise	0.18	45.3%	154	-0.73[-1.35-0.11]
	Dynamic exercise			270	-0.23[-0.62,0.17]
Duration of Continuous Intervention	≤ 3 months	0.81	0%	296	-0.39[-0.98,0.20]
	> 3 months			228	-0.47[-0.73,-0.20]
Timing of Intervention during Pregnancy	Before 20 weeks	0.19	42.3%	402	-0.60[-0.95,-0.25]
	After 20 weeks			126	-0.10[-0.76,0.56]

Following this categorisation, we performed analyses within these subgroups to explore potential variations in the intervention effects. The subgroups allowed us to better understand how different exercise types, intervention durations, and timings during pregnancy may influence the outcomes. The results of these subgroup analyses are presented in the subsequent sections, shedding light on the specific effects of exercise interventions on antenatal depression in pregnant women.

#### 3.5.1 Type of exercise intervention

In this study, we conducted a subgroup analysis on seven papers, classifying them according to the type of exercise intervention. The included studies encompassed three main types of exercise: yoga, categorised as static exercise, and aerobic exercise and HIIT, categorised as dynamic exercise. Our aim was to investigate the effects of these exercise interventions on depressive symptoms in pregnant women.

**Figure 3:**
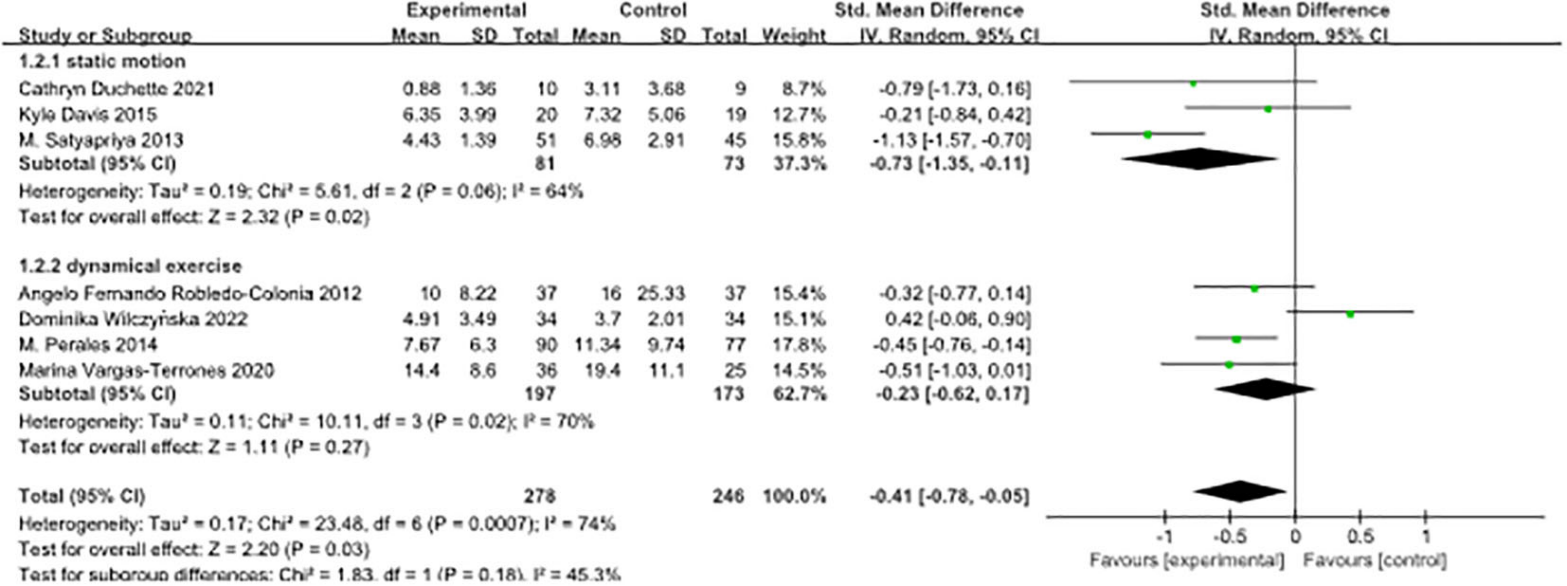
Forest plot of subgroup analyses of pre-pregnancy depression by type of exercise intervention.

The results revealed that both static and dynamic exercise had a significant impact on reducing depressive symptoms in pregnant women. Effect sizes in both groups exhibited moderate heterogeneity, with a standardised mean difference (SMD) of -0.73 (95% confidence interval [-1.35, -0.11]) for static exercise and SMD of -0.23 (95% confidence interval [-0.62, 0.17]) for dynamic exercise. The results of the heterogeneity test demonstrated that the difference between the two subgroups was not statistically significant (p = 0.18). The index of heterogeneity (I²) was calculated at 45.3%, indicating a moderate level of heterogeneity within the studies in each subgroup. For detailed findings and graphical representation, please refer to **Figure 3**.

#### 3.5.2 Duration of continuous intervention

Subgroup analyses were conducted based on the duration of the ongoing intervention. The research literature was divided into two groups, using three months as the cut-off point. Trials lasting less than or equal to three months exhibited a standardised mean difference (SMD) of -0.39 (95% confidence interval [-0.98, 0.20]), while trials lasting more than three months demonstrated an SMD of -0.47 (95% confidence interval [-0.73, -0.20]).

**Figure 4:**
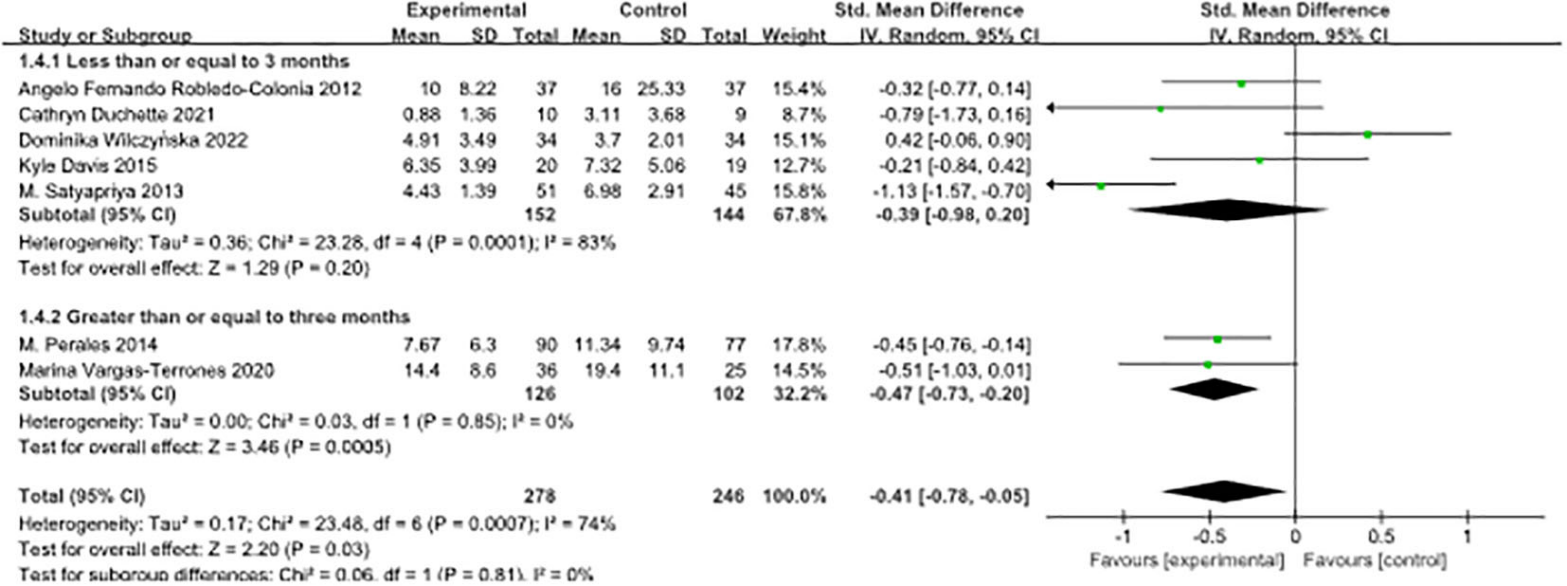
Forest plot of subgroup analyses of pre-pregnancy depression by duration of continuous intervention.

The results of the heterogeneity analysis indicated no statistically significant difference between these two subgroups (P = 0.81). The index of heterogeneity (I²) was calculated at 0%, suggesting no heterogeneity of results within each subgroup. However, given the limited number of studies included, it is essential to interpret these results with caution. To validate these findings and gain a more comprehensive understanding of the effect of exercise intervention duration on outcomes of interest, further studies with larger and more diverse study populations are warranted. For detailed graphical representation, please refer to **Figure 4**.

#### 3.5.3 Timing of intervention during pregnancy

Subgroup analyses were conducted based on the timing of the intervention during pregnancy, and the research literature was divided into two groups using 20 weeks’ gestation as the cut-off point. Trials conducted before 20 weeks of pregnancy showed a standardised mean difference (SMD) of -0.60 (95% confidence interval [-0.95, -0.25]), whereas trials conducted after 20 weeks of pregnancy displayed an SMD of -0.10 (95% confidence interval [-0.76, 0.56]).

**Figure 5:**
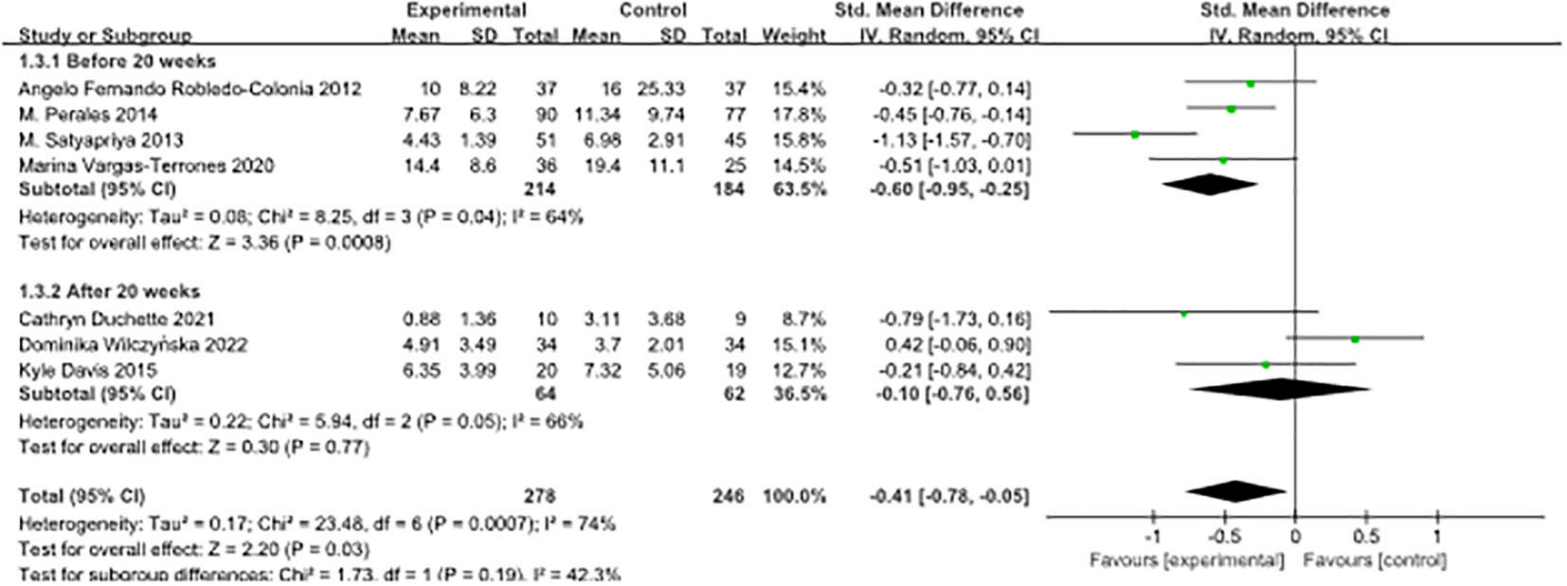
Forest plot of subgroup analyses of pre-pregnancy depression by timing of intervention during pregnancy.

The results of the heterogeneity analysis showed no statistically significant difference between these two subgroups (P = 0.19). The index of heterogeneity (I²) was calculated at 42.3%, indicating moderate heterogeneity of results within each subgroup. These results underscore the potential effectiveness of interventions at different stages of pregnancy in improving the outcomes of interest. However, it is crucial to interpret these findings with caution due to the limited number of included studies. To validate these results and gain a more comprehensive understanding of the effect of the timing of interventions during pregnancy on the outcomes of interest, further studies with larger sample sizes and more diverse study populations are needed, as illustrated in **Figure 5**.

### 3.6 Publication bias test

**Figure 6:**
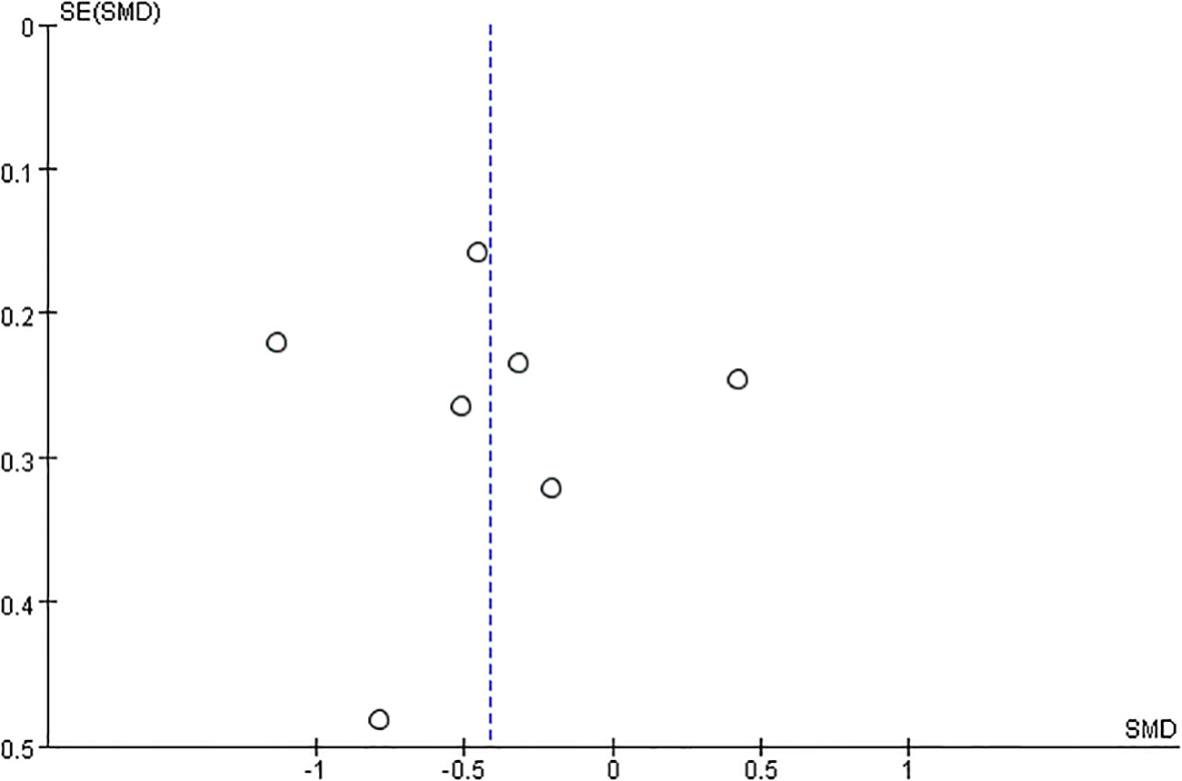
Funnel plot of publication bias tests for included studies.

A funnel plot was meticulously crafted to scrutinise the presence of publication bias within the realm of exercise interventions for antenatal depression. A perusal of the publication bias funnel plot, as depicted in **Figure 6** Funnel plot of publication bias tests for included studies, reveals a harmonious symmetry exhibited across all seven studies. This symmetry, coupled with the absence of conspicuous asymmetrical distortions, signifies a low prevalence of publication bias within the dataset. Additionally, the overall high quality of these studies further reinforces their suitability for inclusion in the ensuing meta-analysis.

## 4 Discussion

### 4.1 Main findings

The meta-analysis clearly showed that exercise intervention had a significant overall effect on reducing antenatal depression symptoms. The standardised mean difference (SMD) of -0.41, 95% confidence interval of -0.78 to -0.05 and p-value of 0.03 confirms the benefit of physical activity in controlling antenatal depression. This result is important because it highlights the potential of exercise as a viable non-pharmacological intervention to alleviate depressive symptoms in pregnant women.

In the context of exercise interventions targeting the amelioration of antenatal depression, the results indicated a notable reduction in depressive symptoms attributable to both types of exercise interventions. Intriguingly, it was observed that static exercise displayed a relatively more substantial effect size, although it did not attain statistical significance (P = 0.18). These findings offer a tantalising suggestion that static exercise may exert a more pronounced influence on moderating outcomes as compared to its dynamic counterpart. It was challenging to directly compare the effects of aerobic exercise and high-intensity interval training (HIIT) interventions due to the limited inclusion of only one article on HIIT. From a physiological perspective, both static and dynamic exercise interventions impact maternal depressive symptoms through endocrine regulatory mechanisms, such as increasing levels of neurotrophic factors, promoting the production of relevant adipocytokines, regulating neurotransmitter expression, enhancing mitochondrial function, and influencing melatonin secretion levels. Furthermore, exercise interventions also influence inflammatory pathways and mediate microRNA expression (37).

However, yoga stands out as a particularly effective intervention due to its emphasis on the mind-body connection and the continuous adjustment and balancing of postures and breathing. These features contribute to restoring mind-body balance, which is crucial in managing antenatal depression. Pregnant women often experience hormonal changes and physical discomfort, and yoga can help them adapt to these physiological and psychological changes. While aerobic exercise and HIIT also offer mental health benefits, certain pregnant women may be unable to engage in high-intensity aerobic exercises due to their physical condition or pregnancy limitations. The gentle movements of yoga are better suited to the physical characteristics of pregnant women, minimising the risk of overexertion that could contribute to antenatal depression. In addressing antenatal depression, the significance of yoga and aerobic exercises extends beyond their biological impacts to include key psychological mechanisms such as emotional regulation, mindfulness, and self-efficacy, which are crucial for the psychological well-being of pregnant women (38). Regular engagement in these activities has been shown to significantly improve mood and reduce stress by increasing the production of mood-enhancing neurotransmitters like endorphins and serotonin, and through practices like controlled breathing in yoga, which amplifies parasympathetic activity and lowers cortisol levels (39). Moreover, yoga’s unique mindfulness practices enhance present awareness and help participants detach from negative thought patterns, reducing depressive symptoms by limiting rumination and anxiety (40). Additionally, achieving fitness goals through yoga and aerobic exercises boosts self-efficacy, providing pregnant women with a sense of mastery over their physical health and better management of mental health challenges This comprehensive approach not only addresses the symptoms of depression but also enhances overall psychological resilience during pregnancy (32).

Ensuring exercise is carried out correctly is vital to prevent any negative effects on antenatal depression. A scientific and well-designed exercise intervention program that prioritizes stimulating the mother’s subjective motivation is essential. It should also avoid high-intensity exercise that may have negative physical and mental impacts on the mother, as well as passive exercises that could add unnecessary pressure. In conclusion, exercise during pregnancy holds promise as an effective intervention for managing antenatal depression. While both static and dynamic exercise interventions offer benefits, the unique features of yoga make it particularly valuable in restoring mind-body balance and addressing the physiological and psychological changes associated with antenatal depression. Selecting appropriate exercise interventions and fostering mothers’ motivation in a carefully designed program are vital for ensuring a positive impact on antenatal depression. Further research and comprehensive studies are needed to delve deeper into the specific effects and mechanisms of various exercise interventions on maternal mental health during pregnancy.

Concerning the temporal dimension of interventions throughout pregnancy aimed at enhancing antenatal depression, the trial outcomes, as unveiled through subgroup analyses, provided insights into the impact of intervention timing. It is worth noting that these interventions, when administered at various points during pregnancy, did indeed manifest an influence on the trial outcomes. Nevertheless, an intriguing observation emerged, with interventions administered prior to the 20th week of gestation exhibiting a comparatively larger effect size, albeit lacking statistical significance (p = 0.19), when juxtaposed with interventions implemented after the 20th week of gestation. This finding implies the possibility of a more conspicuous moderating effect of interventions on the overall outcomes. The presence of moderate heterogeneity within these subgroups may be attributed to variances in the design of the encompassed studies, participant characteristics, or the intricacies of the intervention protocols. The first 20 weeks of gestation represent the early stage of pregnancy, characterised by more pronounced psychological adjustments and mood fluctuations in pregnant women. During this period, the pregnant woman’s body is adapting to hormonal changes and physiological adjustments. Implementing exercise interventions at this stage can effectively help pregnant women cope with psychological stress and mood fluctuations throughout pregnancy, leading to a reduction in symptoms of antenatal depression. Conversely, exercise interventions during the latter half of pregnancy (after 20 weeks of gestation) may be less effective due to increased physical burden and discomfort associated with advancing pregnancy. At this stage, the focus shifts more towards preparing for childbirth, and pregnant women may find it more challenging to actively engage in exercise interventions, potentially reducing the antidepressant effects of such interventions (41).

The outcomes stemming from the subgroup analysis investigating the duration of exercise interventions in the context of antenatal depression treatment shed light on a significant impact evident in both short-term (≤ 3 months) and long-term (> 3 months) interventions. Notably, although both of these categories exhibited substantial effects on the outcomes of interest, the disparity between them failed to attain statistical significance within this subgroup analysis (P = 0.81). More precisely, it was apparent that both short-term and long-term exercise interventions were correlated with enhanced outcomes. These findings may hold substantial implications for the formulation of exercise programs tailored to pregnant women grappling with depressive symptoms, given the observable significant effects on the outcomes of interest for both short-term (≤ 3 months) and long-term (> 3 months) interventions.

In light of the outcomes derived from the three distinct subgroup analyses, it is crucial to acknowledge that these analyses did not yield statistically significant differences. Despite our meticulous examination from various angles, the p-values consistently exceeded the 0.05 threshold, rendering them insufficient to establish the statistical significance of these analyses within the scope of our study. This particular result may be attributed to a confluence of factors, including variances in sample characteristics, the sensitivity of measurement instruments, and methodological heterogeneity. These intricacies may have collectively contributed to our inability to discern noteworthy disparities. It is imperative to recognise that our study possesses certain limitations, encompassing aspects such as data quality, heterogeneity in the criteria for study selection, and the potential influence of publication bias. These limitations bear relevance to our results, necessitating caution in the interpretation of our findings. In light of these constraints, future investigations could embark on a more comprehensive exploration of this domain, delving deeper into potential discrepancies between subgroups. Augmented sample sizes, thorough data collection, and intricate methodological analyses have the potential to furnish a more holistic comprehension of this intricate issue. In summation, our findings underscore the imperative for further inquiries aimed at elucidating the impacts of exercise-based interventions, encompassing exercise type, duration, and timing, on antenatal depression, thereby endeavouring to bridge extant knowledge gaps. The transparency and rigor characteristic of such findings can serve as a compass guiding the trajectory of future research efforts.

These findings have significant implications for healthcare professionals and policymakers in designing exercise intervention programs for pregnant women with antenatal depression. Early and consistent exercise interventions during the first 20 weeks of pregnancy may offer substantial benefits in terms of mental health support and depression management. However, it is essential to recognise the individual differences and preferences of pregnant women when recommending exercise interventions and to ensure that the programs are tailored to meet their specific needs and circumstances.

### 4.2 Strengths and limitations

Our review exhibits several notable strengths. Firstly, the exercise interventions analysed are highly feasible and accessible, as they can be conducted at home or any other convenient location under the guidance of medical professionals, making them practical for pregnant women and their families. Secondly, the inclusion of randomised controlled trials (RCTs) in our review enhances the reliability of our results, as RCTs are considered robust study designs for assessing the efficacy of interventions.

However, certain limitations should be acknowledged in this study. Firstly, the small sample sizes of the groups may limit the generalizability of our results, and thus, the findings should be interpreted with caution. The limited sample size may have reduced the statistical power and precision of our estimates.

Secondly, the lack of blinding in most of the included studies introduces the potential for biases in outcome assessment, which could influence the reliability of the results. Although the issue of blinding in exercise intervention studies remains controversial, it remains an important consideration in the interpretation of the findings.

Thirdly, due to the limited data available, our meta-analysis may not be able to make strong recommendations about the optimal exercise intervention program. The scarcity of comprehensive data in the field of exercise interventions during pregnancy may limit the scope of our analysis and the extent to which we can draw definitive conclusions.

Fourthly, the majority of the included literature primarily focused on analysing depression in the exercise intervention group and the non-intervention group. This narrow focus may have overlooked the potential influence of other factors, such as participants’ physical fitness levels and lifestyle choices, which could confound the inclusion of relevant literature and impact the overall conclusions.

Lastly, the literature reviewed herein predominantly utilised depression measures such as the Beck Depression Inventory II (BDI-II), which were not originally tailored for the perinatal period. This highlights a critical concern: the extent to which these instruments have been modified to accurately reflect the unique psychological and physiological conditions inherent to the perinatal phase. During pregnancy and postpartum, significant changes in a woman’s mental and physical state necessitate specialised assessment tools to ensure precise evaluation and diagnosis of depression. Consequently, there is an imperative need for the development and validation of assessment tools that are specifically designed for the perinatal period. Addressing this gap not only enhances the accuracy of research but also improves clinical outcomes for pregnant and postpartum women, marking a crucial direction for future investigations in perinatal mental health research.

In conclusion, while our review provides valuable insights into the potential benefits of exercise interventions for antenatal depression, it is crucial to recognise the limitations that stem from small sample sizes, lack of blinding, and insufficient data. To build a more robust evidence base and provide comprehensive recommendations, future research should strive to address these limitations by conducting larger-scale and well-designed studies, exploring various aspects of exercise interventions, and taking into account additional influencing factors. By doing so, we can advance our understanding of the role of exercise in managing antenatal depression and optimise its implementation for the overall well-being of pregnant women.

## 5 Conclusion

The main conclusions of this study can be summarised as follows: For the investigation of the intervention effect, we found that exercise has a moderate effect on depression symptoms in pregnant women. The type of exercise intervention, the starting time of the exercise intervention, and the duration of exercise in the exercise intervention programme are the important factors influencing the effect of exercise intervention on antenatal depression; Among the different types of exercise interventions, we found that static exercise, especially yoga, had a significantly better effect on improving antenatal depression than dynamic exercise. Among the different types of exercise interventions, we found that static exercise, especially yoga, had a significantly better effect on improving antenatal depression than dynamic exercise; in terms of the timing of interventions during pregnancy, we found that exercise interventions before 20 weeks’ gestation were significantly more effective than those after 20 weeks’ gestation; and that long-term interventions were slightly more effective than short-term interventions.

It is imperative to underscore the importance of exercising a greater degree of caution in our interpretation of the results, especially when none of the three subgroup analyses have demonstrated statistical significance. We must refrain from making definitive claims regarding the existence of clear associations between specific intervention characteristics and their corresponding outcomes. Instead, we find ourselves compelled to acknowledge the presence of certain limitations within the scope of this study. These limitations encompass a range of factors, including variances in sample characteristics, the sensitivity of measurement tools, variations in study design, and the presence of methodological heterogeneity. It is plausible that these multifaceted elements have played a role in elucidating why the subgroup analyses failed to yield statistical significance. In light of these circumstances, it becomes evident that there is a compelling need for a more comprehensive investigation into the effects of exercise type, duration, and timing on antenatal depression, thereby aiding in the process of bridging the existing gaps in our knowledge. To acquire a more nuanced understanding of the intricate nature of this issue, we extend our encouragement to future research endeavours to consider a broader spectrum of potential influencing factors. This conclusion, framed within the paradigm of cautious and transparent research, not only acts as a guidepost but also propels the trajectory of future research initiatives.

## Data Availability

The original contributions presented in the study are included in the article/**Supplementary Material**. Further inquiries can be directed to the corresponding author.
