# Supplementary material for: The effects of exercise on antenatal depression: a systematic review and meta-analysis
Source: Front Psychiatry. 2024 Sep 23;15:1290418. doi: 10.3389/fpsyt.2024.1290418 (PMC11456520; doi:10.3389/fpsyt.2024.1290418)
Supplement: Supplementary file 1 [file DataSheet1.pdf]

	Random sequence generation (selection bias)	Allocation concealment (selection bias)	Blinding of participants and personnel (performance bias)	Blinding of outcome assessment (detection bias)	Incomplete outcome data (attrition bias)	Selective reporting (reporting bias)	Other bias
Angelo Fernando Robledo-Colonia 2012	+	+	−	+	?	+	?
Cathryn Duchette 2021	+	+	−	?	?	?	?
Dominika Wilczyńska 2022	+	?	?	?	?	+	?
Kyle Davis 2015	+	?	−	+	+	?	?
M. Perales 2014	+	+	−	+	?	+	?
M. Satyapriya 2013	+	+	−	+	?	?	?
Marina Vargas-Terrones 2020	+	+	−	+	+	?	?
