# Supplementary material for: The effects of exercise on antenatal depression: a systematic review and meta-analysis
Source: Front Psychiatry. 2024 Sep 23;15:1290418. doi: 10.3389/fpsyt.2024.1290418 (PMC11456520; doi:10.3389/fpsyt.2024.1290418)
Supplement: Supplementary file 2 [file Table1.docx]

**Pubmed search algorithm**

#1 "Depression"[MeSH Terms]

#2 "depressive symptoms"[Title/Abstract] OR "depressive symptom"[Title/Abstract] OR "symptom depressive"[Title/Abstract] OR "emotional depression"[Title/Abstract] OR "depression emotional"[Title/Abstract]

#3 #1 OR #2

#4 "Exercise"[MeSH Terms]

#5 "Exercises"[Title/Abstract] OR "physical activity"[Title/Abstract] OR "activities physical"[Title/Abstract] OR "activity physical"[Title/Abstract] OR "physical activities"[Title/Abstract] OR "exercise physical"[Title/Abstract] OR "exercises physical"[Title/Abstract] OR "physical exercise"[Title/Abstract] OR "physical exercises"[Title/Abstract] OR "acute exercise"[Title/Abstract] OR "acute exercises"[Title/Abstract] OR "exercise acute"[Title/Abstract] OR "exercises acute"[Title/Abstract] OR "exercise isometric"[Title/Abstract] OR "exercises isometric"[Title/Abstract] OR "isometric exercises"[Title/Abstract] OR "isometric exercise"[Title/Abstract] OR "exercise aerobic"[Title/Abstract] OR "aerobic exercise"[Title/Abstract] OR "aerobic exercises"[Title/Abstract] OR "exercises aerobic"[Title/Abstract] OR "exercise training"[Title/Abstract] OR "exercise trainings"[Title/Abstract] OR "training exercise"[Title/Abstract] OR (("education"[MeSH Subheading] OR "education"[All Fields] OR "Training"[All Fields] OR "education"[MeSH Terms] OR "train"[All Fields] OR "train s"[All Fields] OR "trained"[All Fields] OR "training s"[All Fields] OR "Trainings"[All Fields] OR "trains"[All Fields]) AND "Exercise"[Title/Abstract])

#6 #4 OR #5

#7 "Pregnan"[Title/Abstract] OR "antenatal"[Title/Abstract] OR "ante-natal"[Title/Abstract] OR "ante-partum"[Title/Abstract] OR "prenatal"[Title/Abstract] OR "pre-natal"[Title/Abstract] OR "prepartum"[Title/Abstract] OR "pre-partum"[Title/Abstract] OR "mother"[Title/Abstract] OR "maternal"[Title/Abstract] OR "perinatal"[Title/Abstract] OR "peri-natal"[Title/Abstract] OR "peripartum"[Title/Abstract] OR "peri-partum"[Title/Abstract]

#8 "randomized controlled trial"[Publication Type] OR "randomized"[Title/Abstract] OR "placebo"[Title/Abstract]

#9 #3 AND #6 AND #7 AND #8

**Web of Science search algorithm**

#1 TS=(Depression OR Depressive Symptoms OR Depressive Symptom OR Symptom, Depressive OR Emotional Depression OR Depression, Emotional) and Preprint Citation Index (Exclude – Database)

#2 Exercise, Acute OR Exercises, Acute OR Exercise, Isometric OR Exercises, Isometric OR Isometric Exercises OR Isometric Exercise OR Exercise, Aerobic OR Aerobic Exercise OR Aerobic Exercises OR Exercises, Aerobic OR Exercise Training OR Exercise Trainings OR Training, Exercise OR Trainings, Exercise) and Preprint Citation Index (Exclude – Database)

#3 TS=(Pregnan OR antenatal OR ante-natal OR ante-partum OR prenatal OR pre-natal OR prepartum OR pre-partum OR mother OR maternal OR perinatal OR peri-natal OR peripartum OR peri-partum ) and Preprint Citation Index (Exclude – Database)

#4 TS=(randomized controlled trial OR randomized OR placebo OR RCT ) and Preprint Citation Index (Exclude – Database)

#5 #1 AND #2 AND #3 AND #4 and Preprint Citation Index (Exclude – Database)

**Embase search algorithm**

#1 'depression'/exp OR depression

#2 'depressive symptoms':ab,ti OR 'depressive symptom':ab,ti OR 'symptom, depressive':ab,ti OR 'emotional depression':ab,ti OR 'depression, emotional':ab,ti

#3 #1 OR #2

#4 exercise

#5 'exercises':ab,ti OR 'physical activity':ab,ti OR 'activities, physical':ab,ti OR 'activity, physical':ab,ti OR 'physical activities':ab,ti OR 'exercise, physical':ab,ti OR 'exercises, physical':ab,ti OR 'physical exercise':ab,ti OR 'physical exercises':ab,ti OR 'acute exercise':ab,ti OR 'acute exercises':ab,ti OR 'exercise, acute':ab,ti OR 'exercises, acute':ab,ti OR 'exercise, isometric':ab,ti OR 'exercises, isometric':ab,ti OR 'isometric exercises':ab,ti OR 'isometric exercise':ab,ti OR 'exercise, aerobic':ab,ti OR 'aerobic exercise':ab,ti OR 'aerobic exercises':ab,ti OR 'exercises, aerobic':ab,ti OR 'exercise training':ab,ti OR 'exercise trainings':ab,ti OR 'training, exercise':ab,ti OR 'trainings, exercise':ab,ti

#6 #4 OR #5

#7 'pregnan':ab,ti OR 'antenatal':ab,ti OR 'ante-natal':ab,ti OR 'ante-partum':ab,ti OR 'prenatal':ab,ti OR 'pre-natal':ab,ti OR 'prepartum':ab,ti OR 'pre-partum':ab,ti OR 'mother':ab,ti OR 'maternal':ab,ti OR 'perinatal':ab,ti OR 'peri-natal':ab,ti OR 'peripartum':ab,ti OR 'peri-partum':ab,ti

#8 'randomized controlled trial':ab,ti OR 'randomized':ab,ti OR 'placebo':ab,ti OR 'rct':ab,ti

#9 #3 AND #6 AND #7 AND #8

**Cochrane Library search algorithm**

#1 Depression

#2 (Depressive Symptoms):ab,ti,kw OR (Depressive Symptom):ab,ti,kw OR (Symptom, Depressive):ab,ti,kw OR (Emotional Depression):ab,ti,kw OR (Depression, Emotional):ab,ti,kw

#3 #1 OR #2

#4 exercise

#5 (Exercises):ab,ti,kw OR (Physical Activity):ab,ti,kw OR (Activities, Physical):ab,ti,kw OR (Activity, Physical):ab,ti,kw OR (Physical Activities):ab,ti,kw OR (Exercise, Physical):ab,ti,kw OR (Exercises, Physical):ab,ti,kw OR (Physical Exercise):ab,ti,kw OR (Physical Exercises):ab,ti,kw OR (Acute Exercise):ab,ti,kw OR (Acute Exercises):ab,ti,kw OR (Exercise, Acute):ab,ti,kw OR (Exercises, Acute):ab,ti,kw OR (Exercise, Isometric):ab,ti,kw OR (Exercises, Isometric):ab,ti,kw OR (Isometric Exercises):ab,ti,kw OR (Isometric Exercise):ab,ti,kw OR (Exercise, Aerobic):ab,ti,kw OR (Aerobic Exercise):ab,ti,kw OR (Aerobic Exercises):ab,ti,kw

#6 #4 OR #5

#7 (Pregnan):ab,ti,kw OR (antenatal):ab,ti,kw OR (ante-natal):ab,ti,kw OR (ante-partum):ab,ti,kw OR (prenatal):ab,ti,kw OR (pre-natal):ab,ti,kw OR (prepartum):ab,ti,kw OR (pre-partum):ab,ti,kw OR (mother):ab,ti,kw OR (maternal):ab,ti,kw OR (perinatal):ab,ti,kw OR (peri-natal):ab,ti,kw OR (peripartum):ab,ti,kw OR (peri-partum):ab,ti,kw

#8 (randomized controlled trial):ab,ti,kw OR (randomized):ab,ti,kw OR (placebo):ab,ti,kw OR (RCT):ab,ti,kw

#9 #3 AND #6 AND #7 AND #8
